# Determinants of changes in dietary patterns among Chinese immigrants: a cross-sectional analysis

**DOI:** 10.1186/1479-5868-8-42

**Published:** 2011-05-18

**Authors:** Doenja L Rosenmöller, Danijela Gasevic, Jaap Seidell, Scott A Lear

**Affiliations:** 1 Department of Health Sciences, Free University, Boelelaan 1105, 1081 HV Amsterdam, The Netherlands; 2 Department of Health Sciences, Simon Fraser University, 515 West Hastings Street, Vancouver, BC, V6B 5K3, Canada

## Abstract

**Background:**

Chinese individuals who have immigrated to a Western country initially tend to have a lower risk of cardiovascular disease (CVD) compared to people who are already living there. Some studies have found, however, that CVD risk increases over time in immigrants and that immigration to a western country is associated with changes in dietary patterns. This could have unfavourable effects on the risk of CVD. There is limited knowledge on the food patterns, awareness and knowledge about healthy nutrition among Chinese immigrants. The objective for this study is to explore changes in food patterns, and levels of awareness and knowledge of healthy nutrition by length of residence among Chinese immigrants to Canada.

**Methods:**

120 Chinese individuals born in China but currently living in Canada completed an assessment on socio-demographic characteristics, changes in dietary patterns and variables of awareness and knowledge about healthy foods. With ordinal logistic regression the associations between the quartiles of length of residence and dietary patterns, variables of awareness and knowledge about healthy foods were explored, adjusting for age, sex, education and body mass index.

**Results:**

More than 50% of the participants reported increasing consumption of fruits and vegetables, decreasing the use of deep-frying after immigration. Increased awareness and knowledge about healthy foods was reported by more than 50% of the participants. Ordinal regression indicated that Chinese immigrants who lived in Canada the longest, compared to Chinese immigrants who lived in Canada the shortest, consumed significant greater portion sizes (OR: 9.9; 95% CI: 3.11 - 31.15), dined out more frequently (OR: 15.8; 95% CI: 5.0 - 49.85), and consumed convenience foods more often (OR: 3.5; 95% CI: 1.23 - 10.01).

**Conclusions:**

Chinese immigrants reported some favourable changes in their dietary intake and greater awareness and more knowledge about healthy foods after immigration. However, an increase in portion size, an increased frequency of dining out and an increased consumption of convenience foods could indicate some unfavourable changes. These results suggest that health promotion strategies should build on the observed benefits of improved nutritional knowledge and target areas of portion size and convenience eating.

## Background

Chinese immigrants are a large and increasingly important part of the population in Western countries. In the United States, the Chinese population was the largest sub-group of the Asian population in 2000 [1]. For Canada, it has been predicted that the Chinese population will be the fastest-growing of all of the minority populations [2]. In Europe, the Chinese population is still growing and is the third biggest population amongst non-EU immigrants [3]. Compared to people living in Western countries, Chinese people have lower cardiovascular disease (CVD) rates [4]. Moreover, at the time of immigration, Chinese immigrants tend to be healthier than the native-born population, termed the 'healthy immigrant effect'[5]. However, investigations on Chinese immigrant groups in North America show that this health advantage may diminish over time [4,6]. As Chinese populations are becoming an increasingly significant group in Western countries, identifying their health needs is important for health promotion and disease prevention strategies.

Studies have reported that the prevalence of CVD risk factors is higher among Chinese immigrants in their new country compared to Chinese people living in China [7,8]. Many of these risk factors are influenced by nutrition which subsequently may be influenced by immigrating to a more Western country [9]. Nutrition is of special interest given that it can change towards a healthier lifestyle, for instance by increasing the consumption of fruits and vegetables [10], but it is also a potential risk factor for diseases, for it can increase the risk for obesity and cardiovascular diseases [11]. For these reasons, nutrition is considered a key lifestyle contributor in reducing or causing, chronic diseases [9,12].

Research shows that a difference in food consumption exists between the Chinese population living in China and Chinese immigrants living in a Western country [8,13-15]. People in China generally have a lower intake of foods that are high in fat, and a higher intake of complex carbohydrates and fibres [8,16,17]. Previous studies have reported that Chinese immigrants who immigrated to North America adopted a more Western diet as indicated by an increase in their consumption of grain products, animal products, dairy products, sweets and fats [18,19]. However, there is inconsistency in the results of various studies. For instance, it has been observed that Chinese immigrants actually increase the consumption of foods containing less fat after immigrating to Canada [20].

While previous studies on dietary patterns of Chinese immigrant have mainly focused on changes in dietary behaviour, there is a gap in research regarding the knowledge and awareness that Chinese immigrants have about the availability and affordability of foods in their new country. This is important to understand as dietary guidelines and food labelling differ between the two countries [21]. Past research on these matters is unclear, with one study indicating that Chinese immigrants in North America were not aware of dietary guidelines, food labels or other sources of dietary information [22], another study reported that Chinese immigrant do obtain new knowledge about healthy foods [20]. Since changes in nutrition habits and dietary patterns are usually preceded by knowledge and awareness, understanding these factors in Chinese immigrants is valuable in identifying determinants of dietary patterns and therefore important for formulating future CVD preventative actions among Chinese immigrants in Western countries. In addition, knowledge and dietary patterns may depend on the length of time immigrants have spent in their new country. To date, no study has investigated these issues in a comprehensive fashion. Therefore, the purpose of this research was to explore the change in dietary patterns among individuals of Chinese origin who immigrated to Canada and how these patterns were related to time since immigration. Furthermore, we assessed the knowledge of healthy food choices among Chinese immigrants and their opinion about the availability of healthy foods. The results of this study can be used to inform prevention strategies for appropriate nutrition education programs aimed at Chinese immigrants.

## Methods

The current investigation is a sub-study of the Multi-Cultural Community Health Assessment Trial (M-CHAT). Details on the study and participant recruitment have been published elsewhere [23]. Briefly, the M-CHAT study is a community based study cohort of apparently healthy 30-65 year old men and women. One of the study aims was to describe body fat distribution of individuals from different ethnic backgrounds, including Chinese immigrants. Participants were recruited by a number of methods, including local media advertisements, community events and notices placed at community centres and cultural organizations. There were 217 people of Chinese origin included in the initial study, of which 120 were born in China and had immigrated to Canada after the age of 18. Fluency in English was not a requirement for participation. All participants provided informed consent and this study was approved by the Simon Fraser University Research Ethic Board.

Participants completed a self-administered questionnaire covering items such as age, sex, ethnicity, marital status, educational level and household income. Acculturation was measured as the length of residence in Canada. Length of residence was obtained by subtracting age derived from birth date from self-reported age at the time of arrival in Canada. A comprehensive questionnaire on the knowledge, beliefs and awareness of health, as well as health behaviours, was developed based on the findings of earlier qualitative interviews held with our target communities in combination with previously reported questionnaires. The present cross-sectional investigation focuses on the part of the questionnaire addressing perceived changes in dietary patterns, food preparation, and nutrition knowledge and awareness since immigration. Table 1 shows all questions asked concerning changes in dietary patterns and nutrition knowledge and awareness. All questions, except for portion size, included at least three sub-items, see Table 1. For instance the question 'Has your interest in information about the food you eat, such as ingredients, nutrition information and taking note of food labels, changed since coming to Canada?' included 6 sub-items, for example I look at ingredients in the food I buy, I put effort into making sure the food I buy has good nutritional value, I read the nutritional information table on food products, etc.. All questions were based on a 5-point Likert-scale, ranging from 'much less', to 'no change' to 'much more'. To present the data comprehensibly the frequency results are shown in three categories: increased change/more often/easier (combined responses of 4 and 5), decreased change/less often/harder (combined responses of 1 and 2) and no change (response 3). Length of residence in Canada (time since immigration) was categorized into quartiles (cut off points: 0.2 - 12.0; 12.1 - 18.6; 18.7 - 32.1; 32.2 - 49.9 years).

**Table 1:** Questions asked of participants addressing changes in dietary patterns and nutrition knowledge and awareness

Questions	Sub-items	Answer possibilities (5-point Likert scale)
Has your general portion size (how much you eat in one meal) changed since coming to Canada?	- No sub-items	1. Eat much less 2. 3. No change 4. 5. Eat much more

How has your method of food preparation changed since coming to Canada?	- Stir-frying/BBQ - Baking/grilling food - Boiling food - Deep frying food - Microwaving food - Eating vegetables raw	1. Much less often 2. 3. No change 4. 5. Much more often

How have the foods you generally eat changed since coming to Canada?	- Vegetables - Potatoes, rice - Convenience foods - Fruit - Soft drinks/pop/soda - Dairy products - High fat/fried foods - Deserts/candy/sweets - White meat - Red meat - Restaurant meals/dining out	1. Eat much less 2. 3. No change 4. 5. Eat much more

Has your interest in information about the food you eat, such as ingredients, nutrition information and taking note of food labels, changed since coming to Canada?	- I look at ingredients in the food I buy - I put effort into making sure the food I buy has good nutritional value - I read the nutritional information table on food products - I understand the nutritional information table on food products - I hear about which foods are good for me through the media and advertising	1. Much less often 2. 3. No change 4. 5. Much more often

How do you feel about these issues in Canada compared to your home country?	- Finding fresh fruit and vegetables - Finding low fat food options - Choosing healthy food when dining out	1. Much harder in Canada 2. 3. No Change 4. 5. Much easier in Canada

Data are predominantly descriptive in nature and presented as follows: continuous data were compared using independent t-test, categorical data were compared using Chi-square analyses. For socio-demographics, the data of men and women were compared. All food questions are presented as counts and percentages. The association between variables related to perceived changes in dietary patterns and length of residence were analyzed with Chi-square analyses. The significant outcomes from the Chi-square analyses were subsequently analyzed with ordinal logistic regression analysis, presented with odds ratios (OR) and 95% confidence interval (CI). All ordinal regression analysis were adjusted for age, sex, education and body mass index. The food variables of interest were used as the dependent variable in the original five categories of possible answers ('much less', 'less', 'no change', 'more' and 'much more'). Length of residence was used as a categorical variable expressed as quartiles of length of residence. A p-value of 0.05 was considered as statistically significant. Statistical analyses were performed using Statistical Package for Social Sciences (SPSS) 18.0 (SPSS Inc, Chicago, IL).

## Results

### Socio-demographics

Table 2 summarizes participants' socio-demographic and anthropometric characteristics. Women were on average older than men, while men had higher household incomes compared to women. There were no other statistically significant differences between men and women on remaining socio-demographic characteristics.

**Table 2:** Demographic characteristics

	Male (n = 60)	Female (n = 60)	p-value
Age (years)^a^	50.4 (8.2)	53.3 (7.1)	0.039

Length of residence (years)^b^	17.8 (11.4-28.9)	17.2 (11.7-31.0)	0.5

Married	53 (88.3)	44 (73.3)	0.065

Body Mass Index (BMI, kg/m^2^)^a^	26.2 (3.4)	26.0 (3.6)	NA*

Overweight^c^			
(BMI > 25.0 kg/m^2 ^and ≤ 29.9 kg/m^2^)	26 (44.2)	28 (47.6)	NA*

Obese^c^			
(BMI ≥ 30 kg/m^2^)	10 (17)	9 (15.3)	NA*

Educational level			0.98
Less than high school	7 (11.7)	6 (10.0)	
High school graduation	13 (21.7)	14 (23.3)	
Some post secondary education	4 (6.7)	5 (8.3)	
Post secondary education	25 (41.7)	23 (38.3)	
Post graduate education	11 (18.3)	12 (20.0)	

Household income			0.019
< $ 20.000	4 (6.7)	7 (11.7)	
$ 20.000 - $ 40.000	16 (26.7)	21 (35.0)	
$ 40.000 - $ 60.000	11 (18.3)	17 (28.3)	
>$ 60.000	29 (48.3)	15 (25.0)	

^a^mean (± SD)

^b^Median (25% - 75%)

^c^n(%)

* Not Applicable as men and women were matched for body mass index range.

### Change in dietary patterns

**Figure 1:**
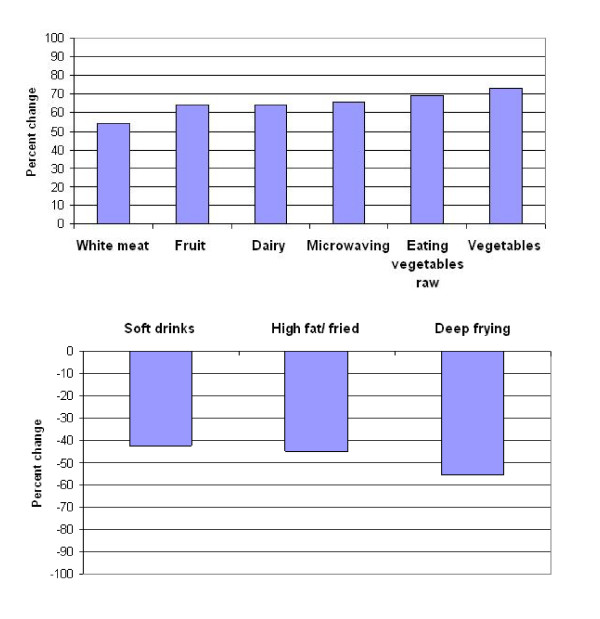
**Percentages of reported changes for dietary preparation and food items**. Food preparation and food items that were reported by more than 50% of participants as increased after immigration based on a response of 4 or 5 on the 5-point Likert scale (top panel). Food preparation and food items that were reported by more than 40% of participants as decreased after immigration based on a response of 1 or 2 on the 5-point Likert scale (top panel).

More than 50% of Chinese immigrants reported an increase in their consumption of fruit and vegetables, white meat and dairy products after immigration (Figure 1). Similarly, when it comes to food preparation, more than 50% of the immigrants reported an increased use of a microwave for cooking as well as eating vegetables raw in a salad or otherwise. More than 50% of the participants reported a decrease in deep-frying food and more than 40% of the participants reported less consumption of high fat/fried foods as well as a decreased consumption of soft drinks (Figure 1). The majority of participants reported no change in their consumption of potatoes/rice after immigration. Similarly, boiling as a form of food preparation was reported by more than 50% of the participants as having remained constant.

**Figure 2:**
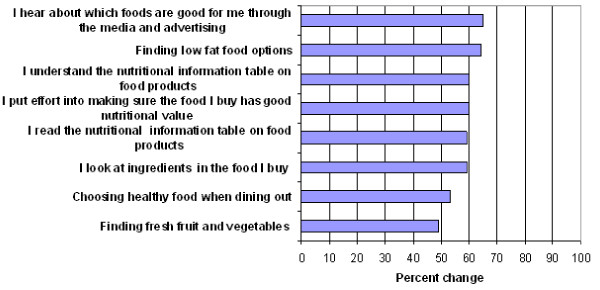
**Reported increased change on knowledge and availability questions**.

Close to 60% of the Chinese immigrants reported reading nutrition information tables on food products more often and understanding the nutrition information tables better after immigration, (Figure 2). More than 60% of the Chinese immigrants reported to have heard more about healthy foods after immigration to Canada through media and advertisements and also found it easier to find low fat food options. Moreover, almost 50% reported that it was easier to find fresh fruits and vegetables after immigration. More than 50% of Chinese immigrants chose healthy foods when dining out and looked at ingredients when buying food.

Using Pearson's Chi-square test, length of residence was significantly associated with portion size (X^2 ^= 22.68 (12), p = 0.031), convenience foods (X^2 ^= 25.07 (12), p = 0.014), restaurant meals/dining out (X^2 ^= 26.01 (12), p = 0.011), and preparing food through boiling (X^2 ^= 21.45 (12), p = 0.044).

The results of ordinal regression revealed that portion sizes increased with length of residence after adjusting for age, sex, education and body mass index. Compared to immigrants from the lowest quartile of length of residence, immigrants from the highest quartile consumed bigger portion sizes (OR: 9.9; 95% CI: 3.11 - 31.15) (Table 3). Restaurant meals were also consumed more often (OR: 15.8; 95% CI: 5.0 - 49.85) by Chinese immigrants within the highest quartile of length of residence compared to the lowest quartile. Furthermore, convenience foods were consumed more often (OR: 10.0; 95% CI: 1.23 - 10.01) by Chinese immigrants within the highest quartile of length of residence compared to the first quartile. Boiling as a way of preparing food was used less often (OR: 0.07; 95% CI: 0.02 - 0.24) by Chinese immigrants within the highest quartile of length of residence compared to the first quartile. All these analysis where adjusted for age, sex, education and body mass index. There were no significant associations between knowledge and awareness of nutrition and foods with length of residence.

**Table 3:** Adjusted odds ratios of Length of residence (quartiles) with 4 food variables

	Odds Ratio^a^	95% CI	p-Value
**Portion size**			

Length of residence (years):	Reference		

< 12.0	2.6	0.96 - 6.82	0.06

12.1 - 18.6	2.9	1.89 - 7.63	0.04

18.7 - 32.1	9.9	3.11 - 31.15	<0.001

> 32.2			

**Restaurant meals/dining out**			

Length of residence (years):	Reference		

< 12.0	2.7	1.05 - 6.91	0.04

12.1 - 18.6	5.9	2.23 - 15.82	< 0.001

18.7 - 32.1	15.8	5.00 - 49.85	< 0.001

> 32.2			

**Convenience foods**			
Length of residence (years):	Reference		
< 12.0	1.8	0.70 - 4.37	0.2
12.1 - 18.6	2.9	1.16 - 7.41	0.02
18.7 - 32.1	3.5	1.23 - 10.01	0.02
> 32.2			

**Boiling**			
Length of residence (years):	Reference		
< 12.0	0.3	0.11 - 0.82	0.02
12.1 - 18.6	0.3	0.09 - 0.75	0.01
18.7 - 32.1	0.07	0.02 - 0.24	< 0.001
> 32.2			

^a^Adjusted for gender, age, education and body mass index

## Discussion

Our results indicate that after immigration to Canada, the majority of Chinese immigrants tended to consume more healthy foods like fruits and vegetables, and showed a decrease in less healthy food habits such as deep-frying as a way of preparing foods. Furthermore, as a whole, Chinese immigrants reported a higher awareness of healthy food choices and increased knowledge of nutritional information on food tables and food products after immigration, however, there was no association between these variables and length of residence. Length of residence did show a positive correlation to potentially less healthy food habits such as portion size, restaurant meals/dining out and convenience foods after immigration of Chinese immigrants to Canada. Taking into account that CVD risks are higher among Chinese immigrants after immigration [7,8], this trend could partially be explained by the increase in portion sizes, more frequent dining out after immigration and higher consumption of convenience foods.

Our findings are consistent with other studies that reported an increase in consumption of fruits and vegetables after immigration in Chinese immigrants [18,19,24]. This is supported by our study population reporting an increased awareness of healthy foods, and greater than 50% reporting availability of fruits and vegetables. An earlier study reported a greater year-round availability of fresh fruits and vegetables in supermarkets in Canada as opposed to China [24]. In addition, an increased consumption of fruits and vegetables could also be related to how these products are displayed in supermarkets. Research shows that with more shelf-space for fruits and vegetables the purchase and consumption increases [25]. It is possible that fruits and vegetables are displayed differently in shops in Western countries compared to China.

While our results are in line with those of Kwok et al. who reported a decrease in fat intake by reducing the consumption of fried foods among Chinese immigrants [20], others reported an increase in the consumption of fatty foods in Chinese immigrants after immigration to a Western country [18,19]. This dissimilarity in outcome could be related to differences in the investigated populations. According to Osypuk et al. neighbourhoods with a higher proportion of Chinese immigrants tended to have a diet lower in fat [26], suggesting that Chinese immigrants preserve their traditionally healthier food practices better when living in these neighbourhoods, whereas neighbourhoods with a lower proportion of people of Chinese background may be less likely to have foods available familiar to the Chinese immigrants. This study was conducted in Vancouver and its surrounding areas, where throughout the city, neighborhoods can be found with a high proportions of Chinese immigrants, which may have resulted in a greater availability of familiar foods. However, the previous cited studies also were conducted in neighbourhoods of predominantly Chinese immigrants. It is possible that our different findings may be due to how the data were collected. Previous studies used food frequency questionnaires [18-20], while our questionnaire was based on participant perceptions of change.

The suggested increase in portion size after immigration to Canada described in our study has, to our knowledge, not been investigated in previous research. In Canada, as well as in China, nutrition guidelines are used to recommend people about healthy amounts of food consumed daily. Although the nutrition guidelines are presented differently in the two countries, similar amounts of food are recommended [21]. Therefore it is not likely that differences in nutritional guidelines has an influence on the change in portion size among Chinese immigrants after immigrating to Canada. A possible explanation for the larger portion sizes could be that larger portion sizes are offered in shops and restaurants. Additionally, Whalqvist reported that when new foods are included along with the availability of traditional foods, this increase in variety can result in an increase in food consumption [11].

Consistent with previous research [27], our study revealed a higher awareness of healthy food habits among Chinese immigrants after immigration. Food labels were reported to be read more often by Chinese immigrants after immigration, and were understood better. However, this change was not found to be related to length of residence. One explanation could be that these changes occur soon after immigration to Canada. As for better understanding and more frequent reading of food labels, a possible explanation could lie in the different requirements concerning regulation of food labelling in China versus Canada. Chinese regulations only require a list of ingredients, and the net weight (the weight of the product alone) to be present on pre-packaged food products [28]. Canadian regulations require the same information on a food label, along with a table of nutrition facts given per serving [29]. Moreover, four diet-related health claims are allowed to be shown on Canadian pre-packaged foods suitable for diets that reduce risk factors for chronic diseases or conditions. Research has shown that these health claims improve the quality of dietary choices [30].

Our research indicates that Chinese immigrants increased their portion size, dining out and consumption of convenience foods after immigration to Canada. As we did not examine the actual composition of the foods which were eaten as part of the portion sizes, dining-out meals and convenience foods, we cannot say if this is a healthy or unhealthy change. An increase in portion size may be interpreted as an unhealthy change for it increases the risk of obesity [31,32]. However, portion sizes of fruits and vegetables could also have increased, which would be beneficial for the risk of CVD [10]. We also found that more than 50% of the Chinese immigrants reported that it was easier to choose healthy foods when dining out after immigration. In an earlier investigation we reported that length of residence was related to household income [6] and this may in part explain the greater reported frequency of dining out with increased length of residence as those people with greater income may have more financial freedom to eat in restaurants. Lastly, more than 60% of Chinese immigrants reported hearing more about healthy food choices through the media after immigration which may relate to eating more healthy foods. However, in these same participants, we reported that length of residence was positively associated with increased thickness of the carotid artery, a known precursor to CVD, and this was also greater than non-immigrants of the same age [6]. It is possible that despite a number of reported favourable changes in dietary patterns in this group, these benefits may be outweighed by the negative health effects of increased portion sizes as well as increased consumption of convenience foods and restaurant meals.

Several limitations should be considered when interpreting our results. Our study participants tended to have a high level post-secondary education and household income which has been found to be associated with a greater health outcomes and knowledge [33]. In addition, the M-CHAT study purposely recruited participants across a range of BMI values and therefore oversampled for Chinese people who were obese. The greater number of people with obesity in our study population may be reflected in our findings that portion sizes increase with length of residence in Canada, as portion sizes tend to be higher in people with higher BMI. However, our earlier study did not find a relationship between increasing BMI and length of residence [6]. As we did not assess dietary intake but rather perception of dietary changes with immigration, we cannot be certain of the exact changes in diet, and actual changes in dietary behaviours with immigration should be explored in future research. We must also acknowledge that recall of previous dietary patterns has been found to be less accurate than the recall of current dietary patterns [22]. Finally, as this was a cross-sectional study, longitudinal data are needed to explore whether there is a causal relationship between the change in dietary patterns and CVD risk among Chinese immigrants.

## Conclusions

Our study provided a novel comprehensive investigation of dietary patterns as well as knowledge and awareness of healthy eating following immigration. We found that Chinese immigrants in this study reported adopting a number of healthy food habits such as increased fruits and vegetables, and cooking methods associated with healthier food preparation after immigrating to Canada. This was consistent with the observation that more than 50% of the participants reported a greater knowledge and awareness of healthy foods suggesting that current initiatives for promoting improved nutrition are having some effect at educating this population. However, participants also reported an increase in portion size, an increased frequency of dining out and an increased consumption of convenience foods, and these factors increased significantly as time since immigration increased. Based on these results, health promotion and disease prevention strategies should focus on reinforcing the good dietary patterns while emphasizing the importance of the possible unhealthy change of consuming bigger portion sizes, dining out more frequently and consuming more convenience foods upon immigration.

## List of abbreviations

BMI: Body mass index; CI: Confidence interval; CVD: Cardiovascular Diseases; M-CHAT: Multi-Cultural Community Health Assessment Trial; OR: Odds ratios;

## Competing interests

The authors declare that they have no competing interests.

## Authors' contributions

DLR carried out the literature study, the statistical analysis and contributed to draft the manuscript. DG participated in its design of the study, coordinated and helped to draft the manuscript. JS contributed with the literature study, the design of the study and helped to draft the manuscript. SAL conceived of the study, coordinated the data collection, helped to draft the manuscript and coordination. All authors have read and approved of the final manuscript as submitted.
